# Worldwide Distribution of the *MYH9* Kidney Disease Susceptibility Alleles and Haplotypes: Evidence of Historical Selection in Africa

**DOI:** 10.1371/journal.pone.0011474

**Published:** 2010-07-09

**Authors:** Taras K. Oleksyk, George W. Nelson, Ping An, Jeffrey B. Kopp, Cheryl A. Winkler

**Affiliations:** 1 Department of Biology, University of Puerto Rico at Mayagüez, Mayagüez, Puerto Rico; 2 Laboratory of Genomic Diversity, SAIC-Frederick, Frederick, Maryland, United States of America; 3 Kidney Disease Section, National Institute of Diabetes and Digestive and Kidney Diseases, National Institutes of Health, Bethesda, Maryland, United States of America; University of Utah, United States of America

## Abstract

*MYH9* was recently identified as renal susceptibility gene (OR 3–8, p<10^−8^) for major forms of kidney disease disproportionately affecting individuals of African descent. The risk haplotype (E-1) occurs at much higher frequencies in African Americans (≥60%) than in European Americans (<4%), revealing a genetic basis for a major health disparity. The population distributions of *MYH9* risk alleles and the E-1 risk haplotype and the demographic and selective forces acting on the *MYH9* region are not well explored. We reconstructed *MYH9* haplotypes from 4 tagging single nucleotide polymorphisms (SNPs) spanning introns 12–23 using available data from HapMap Phase II, and by genotyping 938 DNAs from the Human Genome Diversity Panel (HGDP). The E-1 risk haplotype followed a cline, being most frequent within sub-Saharan African populations (range 50–80%), less frequent in populations from the Middle East (9–27%) and Europe (0–9%), and rare or absent in Asia, the Americas, and Oceania. The fixation indexes (F_ST_) for pairwise comparisons between the risk haplotypes for continental populations were calculated for *MYH9* haplotypes; F_ST_ ranged from 0.27–0.40 for Africa compared to other continental populations, possibly due to selection. Uniquely in Africa, the Yoruba population showed high frequency extended haplotype length around the core risk allele (C) compared to the alternative allele (T) at the same locus (rs4821481, iHs = 2.67), as well as high population differentiation (F_ST(CEU vs. YRI)_ = 0.51) in HapMap Phase II data, also observable only in the Yoruba population from HGDP (F_ST_ = 0.49), pointing to an instance of recent selection in the genomic region. The population-specific divergence in *MYH9* risk allele frequencies among the world's populations may prove important in risk assessment and public health policies to mitigate the burden of kidney disease in vulnerable populations.

## Introduction

A genome wide admixture linkage scan followed by fine mapping recently identified *MYH9*, encoding non-muscle myosin heavy chain IIA, as a major susceptibility locus for focal segmental glomerulosclerosis (FSGS), HIV-associated collapsing glomerulosclerosis, also called HIV-associated nephropathy (HIVAN), and end stage kidney disease (ESKD) attributed to hypertension [1], [2]. A series of subsequent studies have confirmed and extended the initial findings for non-diabetic ESKD to a possible role in diabetic ESKD —the leading cause of kidney failure [3]. It has long been noted that African ancestry populations (e.g., African Americans) are more likely to develop kidney disease and have a poorer prognosis than their European descent counterparts. Family clustering of disparate etiologies of kidney diseases has also been reported in African American families [4]. In the United States, African Americans have approximately 3–4-fold higher rates of ESKD compared to European Americans [5]. The risk of HIVAN is 18-fold or greater in African Americans compared to non-African descent populations and it is estimated that the life-time risk of HIVAN among African Americans with HIV-1 disease, in the absence of anti-retroviral therapy, is 10% [6]. *MYH9* provides a plausible genetic explanation for much of this disparity as the *MYH9* alleles and the haplotype most strongly associated with kidney disease are highly frequent in African Americans (allele frequencies ≈60%) and infrequent in European Americans (≤4%)[1]. These studies did not address global distribution of *MYH9* risk alleles, and the historical reasons for this health disparity remained elusive.

Although many *MYH9* SNPs were found to significantly associate with HIVAN and FSGS, any of the three highly correlated SNPs, rs4821480, rs2032487, and rs4821481 in intron 23 plus rs3752462 in intron 13, defined an extended (E) haplotype that was more informative than any single SNP for association with kidney disease [1], [7]. The *MYH9* E-1 haplotype was associated with HIVAN, FSGS, and non-diabetic ESKD (OR = 2.8, 5, 7, p<10^−8^) [1]. The extended haplotype spans 14.9 kb, extending across two haplotype blocks that encompass introns 12–23. All of the *MYH9* single nucleotide polymorphisms (SNPs) most strongly associated with kidney disease fall within this extended block [1]. The *MYH9* E-1 haplotype explains nearly all of the excess burden of major forms of kidney disease in African Americans; for example, the attributable risks are 100% and 70% for HIVAN and FSGS, respectively. The association of *MYH9* risk alleles with HIVAN is particularly worrisome for sub-Sahara Africa where risk alleles are predicted to be at high frequency and more than 22 million adults and children are infected with HIV-1.

In this study, we present an analysis of the E haplotype block and tagging SNPs in a worldwide population survey of major continental populations using a compilation of data from the International HapMap Project and the Human Genome Diversity Panel (HGDP). We analyzed SNPs using data available from HapMap Phase II [8] and HGDP population [9], [10], and genotyped additional SNPs in the HGDP. We used the combined information to reconstruct E haplotypes associated with kidney disease to determine the worldwide distribution and frequencies of risk and protective haplotypes to assess public health implications, especially in settings of high HIV prevalence. A secondary goal was to determine if highly divergent allele frequencies were generated by selection on *MYH9* or by neutral mechanisms. We discussed the observed diversity in the context of local adaptation and population histories.

## Results and Discussion

To determine the worldwide distribution and evolutionary history of non-muscle myosin IIA heavy chain gene (*MYH9*) alleles and the E haplotypes associated with kidney disease in the African American population [1], [2], we obtained genotypes for the Human Genome Diversity Panel comprising DNA from 938 individuals representing 51 unique populations for the three SNPs defining the E haplotype block: SNP rs4821481 (T/c) was available from the CEPH database [8], [9], and SNPs rs4821480 (T/g), and rs3752462 (C/t) were genotyped in-house. SNP rs2032487 which is in near absolute LD with both rs482180 and rs48321481 was not included. To reconstruct the worldwide distribution of the E haplotypes, the haplotypes were inferred using three E haplotype-defining SNPs separately in the 51 distinct ethnic groups from HGDP with 95–100% accuracy.

As shown in Table 1, African populations display the highest heterozygosity compared to all other worldwide populations. Of the three SNPs, rs3752462 is the most variable, particularly in populations outside of Africa that showed the widest range in heterozygosity. The two other SNPs are variable among African populations, and are often fixed in populations east from the Fertile Crescent: Central and East Asia, Oceania, and the Americas (Table 1). The populations in Europe and the Middle East also show high variation at these loci (heterozygosity between 0.1–0.4). These populations show divergence from other African populations for many genetic markers in HGDP [11]. Low heterozygosity values in Yoruba compared to other African populations may be a consequence of selection, but should only be interpreted in conjunction with other indicators of selective sweep [12].

**Table 1:** Observed heterozygosity (Het) and the Ancestral Allele Frequency (P_ANC_)^1^ of SNPs included in the risk and protective *MYH9* haplotypes.

Human Genome Diversity Panel		rs4821480		rs4821481		rs3752462		
(HGDP)		(t/G)		(t/C)		(C/t)		
Region	Population	Het	P_ANC_	Het	P_ANC_	Het	P_ANC_	Total
**Africa**	Bantu-Kenya	0.333	0.667	0.333	0.667	0	0.167	12
	Bantu-Sa	0.375	0.687	0.5	0.625	0.375	0.188	8
	Biaka	0.265	0.779	0.375	0.75	0.265	0.162	36
	Mandenka	0.318	0.795	0.458	0.729	0.25	0.125	24
	Mbuti	0.267	0.533	0.333	0.5	0.4	0.2	15
	San	0.714	0.643	0.714	0.643	0.286	0.143	7
	Yoruba	0.167	0.875	0.24	0.8	0.12	0.1	25
**Total**		**0.295**	**0.746**	**0.382**	**0.703**	**0.232**	**0.148**	**127**
**America**	Colombian	0	0	0	0	0.769	0.538	13
	Karitiana	0	0	.	.	0.435	0.217	24
	Maya	0.045	0.023	0	0	0.32	0.64	25
	Pima	0	0	0	0	0.32	0.76	25
	Surui	0	0	.	.	0.45	0.225	20
**Total**		**0.01**	**0.005**	**0**	**0**	**0.425**	**0.486**	**107**
**South-Central Asia**	Balochi	0.2	0.14	0.2	0.167	0.48	0.36	25
	Brahui	0.12	0.06	.	.	0.56	0.52	25
	Burusho	0	0	0	0	0.542	0.479	25
	Dai	0	0	.	.	0.222	0.111	10
	Hazara	0.043	0.022	.	.	0.565	0.326	25
	Kalash	0.083	0.042	0.056	0.028	0.44	0.46	25
	Makrani	0.16	0.08	.	.	0.52	0.54	25
	Pathan	0.08	0.04	.	.	0.625	0.521	25
	Sindhi	0.125	0.062	.	.	0.6	0.38	25
	Uygur	0	0	0	0	0.333	0.5	10
**Total**		**0.093**	**0.051**	**0.08**	**0.06**	**0.519**	**0.437**	**220**
**East Asia**	Cambodian	0	0	0	0	0.364	0.182	11
	Chinese	0	0	.	.	0.356	0.222	45
	Daur	0	0	0	0	0.4	0.2	10
	Hezhen	0	0	.	.	0.3	0.35	10
	Japanese	0	0	.	.	0.448	0.259	31
	Lahu	0	0	0	0	0.333	0.167	10
	Miaozu	0	0	.	.	0.3	0.15	10
	Mongolian	0	0	0	0	0.625	0.313	10
	Naxi	0	0	.	.	0.3	0.15	10
	Oroqen	0	0	.	.	0.111	0.167	10
	She	0	0	.	.	0.556	0.278	10
	Tu	0	0	.	.	0.3	0.25	10
	Tujia	0	0	.	.	0.4	0.2	10
	Xibo	0	0	.	.	0.111	0.056	9
	Yakut	0	0	0	0	0.52	0.34	25
	Yizu	0	0	0	0	0.444	0.222	10
**Total (East Asia)**		**0**	**0**	**0**	**0**	**0.381**	**0.231**	**230**
**Europe**	Adyghei	0.063	0.031	0.071	0.036	0.412	0.559	17
	Basque	0.136	0.068	0.083	0.042	0.381	0.714	24
	French	0.107	0.054	.	.	0.552	0.655	29
	Italian	0.154	0.077	.	.	0.538	0.5	14
	Orcadian	0.188	0.094	.	.	0.533	0.667	16
	Russian	0.091	0.045	0.077	0.038	0.4	0.64	25
	Sardinian	0.143	0.071	.	.	0.259	0.611	28
	Tuscan	0	0	.	.	0.25	0.75	8
**Total**		**0.123**	**0.061**	**0.075**	**0.037**	**0.414**	**0.627**	**161**
**The Middle East**	Bedouin	0.277	0.245	0.277	0.266	0.292	0.417	49
	Druze	0.146	0.094	0.163	0.105	0.479	0.615	48
	Mozabite	0.241	0.19	0.267	0.2	0.5	0.517	30
	Palestinian	0.235	0.137	0.346	0.212	0.5	0.55	51
**Total**		**0.223**	**0.163**	**0.253**	**0.195**	**0.438**	**0.526**	**178**
**Oceania**	Melanesian	0	0	0	0	0.045	0.023	22
	Papuan	0	0	0	0	0	0	17
**Total**		**0**	**0**	**0**	**0**	**0.026**	**0.013**	**39**

1 The ancestral allele is indicated in capital letters, the allele with >50% in Europeans (major allele) is the first one of the pair.

**Figure 1:**
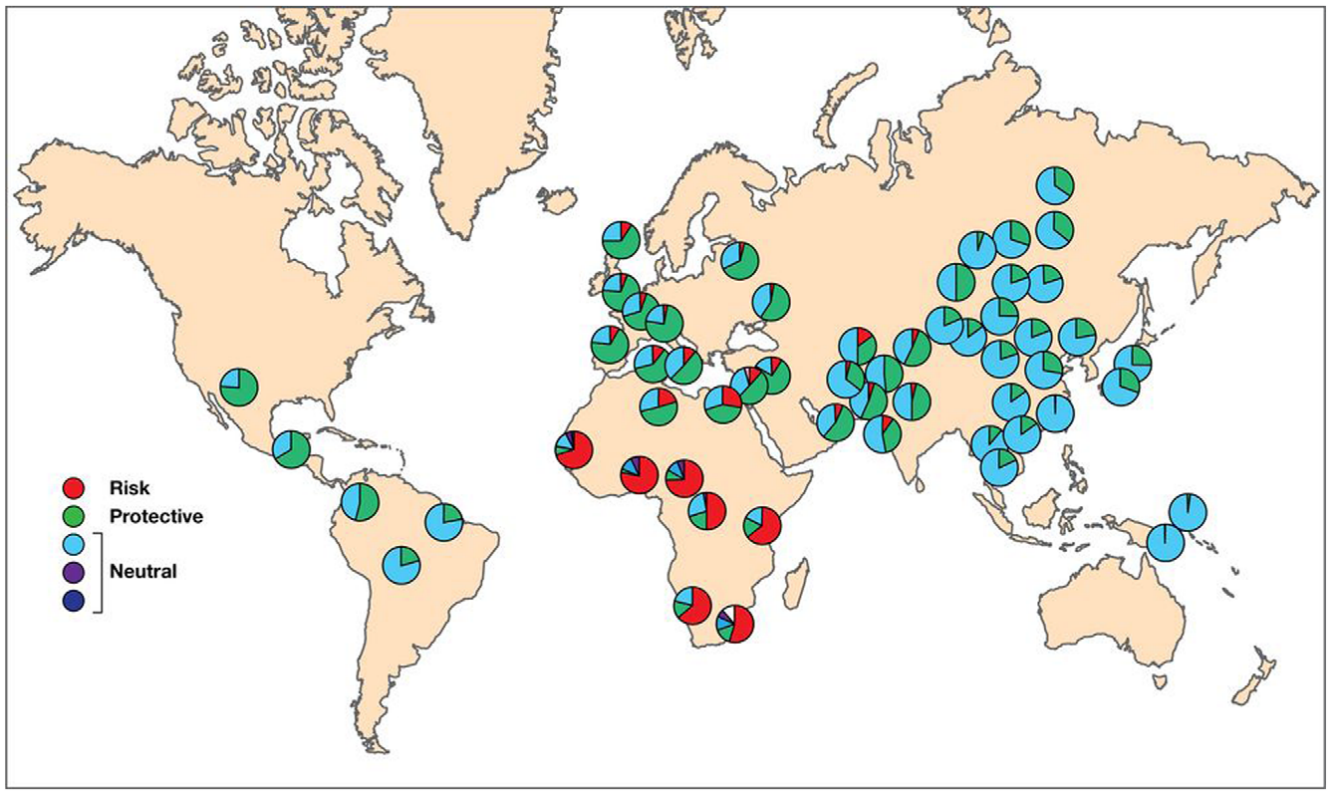
The distribution of *MYH9* haplotypes in the world populations from the HGDP. The haplotype proportions represented by the pie charts can also be found in Table 2.

We found substantial differentiation in the frequencies of rs4821481, as indicated by the increased F_ST_ in the HapMap samples (F_ST(CEU vs. YRI)_ = 0.51)[13], and the HGDP (F_ST_ = 0.49)[11](Figure 1, Table S1, Table S2, Table S3). This single allele can serve as a proxy for the risk haplotype (E-1), since the C allele is present in over 99% of the risk E-1 haplotype (Table 2); the only other haplotype carrying the C allele at rs4821481 is the rare E-5, which was observed in only two of the HGDP populations, with frequencies of 0.02 (Mandenka) amd 0.01 (Palestinian). The reported value of divergence for this allele is considered highly significant and lays within the top 5% of F_ST_ among all SNPs genotyped in the HapMap project [8], [14]–[16]. For instance, the pairwise continental F_ST_s range from CEU vs. CHB+JPT, with the lowest level of differentiation (average F_ST_ = 0.07), to YRI vs. CHB+JPT with the highest (average F_ST_ = 0.12)[17], while the differences in the where pair-wise comparisons between France, Palestine, Han and Yoruba in the Human Diversity Panel all show F_ST_ <0.15 [11], [18].

**Table 2:** Worldwide distribution of *MYH9* risk haplotypes in the HGDP samples.

Regions/Populations	E-1‡(GcCT)	E-2(TtTC)	E-3(TtTT)	E-4(GtTC)	E-5 (GcTC)	other†	count
**Africa**	0.69	0.09	0.15	0.05	.	0.01	253.8
Bantu-Kenya	0.67	0.17	0.17	.	.	.	24
Bantu-Sa	0.56	0.13	0.13	0.06	.	0.13	16
Biaka	0.74	0.10	0.11	0.06	.	.	72
Mandenka	0.71	0.06	0.17	0.04	0.02	.	48
Mbuti	0.50	0.17	0.30	0.03	.	.	30
San	0.64	0.14	0.21	.	.	.	14
Yoruba	0.80	0.02	0.10	0.08	.	.	49.8
**America**	**.**	**0.49**	**0.51**	**.**	**.**	**.**	**212**
Colombian	.	0.54	0.46	.	.	.	26
Karitiana	.	0.21	0.79	.	.	.	48
Maya	.	0.67	0.33	.	.	.	48
Pima	.	0.76	0.24	.	.	.	50
Surui	.	0.23	0.78	.	.	.	40
**South Central Asia**	**0.05**	**0.44**	**0.51**	**.**	**.**	**.**	**435.7**
Balochi	0.14	0.36	0.50	.	.	.	50
Brahui	0.06	0.52	0.42	.	.	.	50
Burusho	.	0.48	0.52	.	.	.	50
Dai	.	0.10	0.90	.	.	.	20
Hazara	0.02	0.33	0.65	.	.	.	46
Kalash	0.04	0.46	0.50	.	.	.	50
Makrani	0.08	0.54	0.38	.	.	.	50
Pathan	0.04	0.52	0.44	.	.	.	50
Sindhi	0.06	0.38	0.55	.	.	0.01	49.8
Uygur	.	0.50	0.50	.	.	.	20
**East Asia**	**.**	**0.23**	**0.77**	**.**	**.**	**.**	**455.9**
Cambodian	.	0.18	0.82	.	.	.	22
Chinese	.	0.22	0.78	.	.	.	90
Daur	.	0.20	0.80	.	.	.	20
Hezhen	.	0.35	0.65	.	.	.	20
Japanese	.	0.26	0.74	.	.	.	58
Lahu	.	0.15	0.85	.	.	.	20
Miaozu	.	0.15	0.85	.	.	.	20
Mongolian	.	0.30	0.70	.	.	.	20
Naxi	.	0.15	0.85	.	.	.	19.9
Oroqen	.	0.20	0.80	.	.	.	20
She	.	0.28	0.72	.	.	.	18
Tu	.	0.25	0.75	.	.	.	20
Tujia	.	0.20	0.80	.	.	.	20
Xibo	.	0.06	0.94	.	.	.	18
Yakut	.	0.34	0.66	.	.	.	50
Yizu	.	0.20	0.80	.	.	.	20
**Europe**	**0.06**	**0.62**	**0.31**	**.**	**.**	**.**	**325.5**
Adyghei	0.03	0.56	0.41	.	.	.	34
Basque	0.08	0.69	0.23	.	.	.	47.9
French	0.05	0.66	0.29	.	.	.	57.9
Italian	0.08	0.51	0.41	.	.	.	27.8
Orcadian	0.09	0.66	0.25	.	.	.	32
Russian	0.04	0.64	0.32	.	.	.	50
Sardinian	0.07	0.61	0.32	.	.	.	55.9
She	.	.	1	.	.	.	2
Tuscan	.	0.75	0.25	.	.	.	16
**The Middle East**	**0.17**	**0.52**	**0.30**	**.**	**.**	**.**	**355.7**
Bedouin	0.27	0.43	0.30	.	.	.	97.9
Druze	0.09	0.61	0.29	.	.	.	96
Mozabite	0.20	0.52	0.28	.	.	.	60
Palestinian	0.12	0.54	0.33	.	0.01	.	101.8
**Oceania**	**.**	**0.01**	**0.99**	**.**	**.**	**.**	**78**
Melanesian	.	0.02	0.98	.	.	.	44
Papuan	.	.	1	.	.	.	34
**HGDP Total**	**0.13**	**0.38**	**0.48**	**0.01**	**<0.001**	**<0.001**	**2116.7** †

‡ The haplotypes are composed of SNP loci of the *MYH9* gene: rs4821480 (T/g), rs4821481 (T/c), rs2032487 (C/t), and rs3752462 (C/t) in that order. We genotyped rs4821480 and rs2032487, used rs4821481 from the previously published data (HGDP), and always inferred rs2032487 (in the lower case).

† Haplotypes with very low probabilities (P<0.001) have been ignored.

Although the divergence between the two groups is suggestive of selection, F_ST_ values alone are insufficient for determining if a locus is the target of selection, because high individual values of F_ST_ could also result from genetic drift or demographic events [12]. We expanded the comparison to the inferred E haplotypes for pairwise F_ST_ scores between continental populations (Table 3). Most of these calculations yield statistically significant values >0.25 (except Europe vs. South-Central Asia), but F_ST_ values for haplotypes are markedly lower than those calculated from individual SNPs. The largest population differences were observed between African population and non-African populations (F_ST_ = 0.27–0.4, Table 3); the difference in frequencies between European and East Asian populations was also elevated (F_ST_ = 0.23, Table 3). Within continents, most population comparisons do not yield large differentiation estimates (Table S1, Table S2, Table S3). Indeed, only one of these comparisons was noteworthy (Yoruba vs. Bantu from South Africa (BANTU-SA), F_ST_ = 0.37)(Table S1). *MYH9* harbors SNPs with some of the highest F_ST_ values on chromosome 22: F_ST_ (CEU vs. YRI)  = 0.5–0.65 in *MYH9* (expected chromosome-wide F_ST_ = 0.29)[13]. The existence of high values for multilocus or haplotype Fst values across regions can still be considered as a starting point in identifying selective targets [1], [19].

**Table 3:** Geographic differentiation between continental populations estimated by pairwise F_ST_-s with loci representing risk and protective *MYH9* haplotypes†.

Continent	1	2	3	4	5	6	7
1. Africa		+	+	+	+	+	+
2. Middle East	0.27		+	+	+	+	+
3. Europe	0.37	0.01		+	+	+	+
4. East Asia	0.38	0.19	0.23		+	+	+
5. South-Central Asia	0.32	0.04	0.05	0.07		—	+
6. America	0.39	0.06	0.07	0.08	0.01		+
7. Oceania	0.40	0.29	0.37	0.04	0.18	0.23	

† Conventional pairwise F_ST_ values were computed directly from haplotype frequencies in Arlequin [39]. The top triangle in the matrix shows significance of the given comparison (p<0.05).

**Figure 2:**
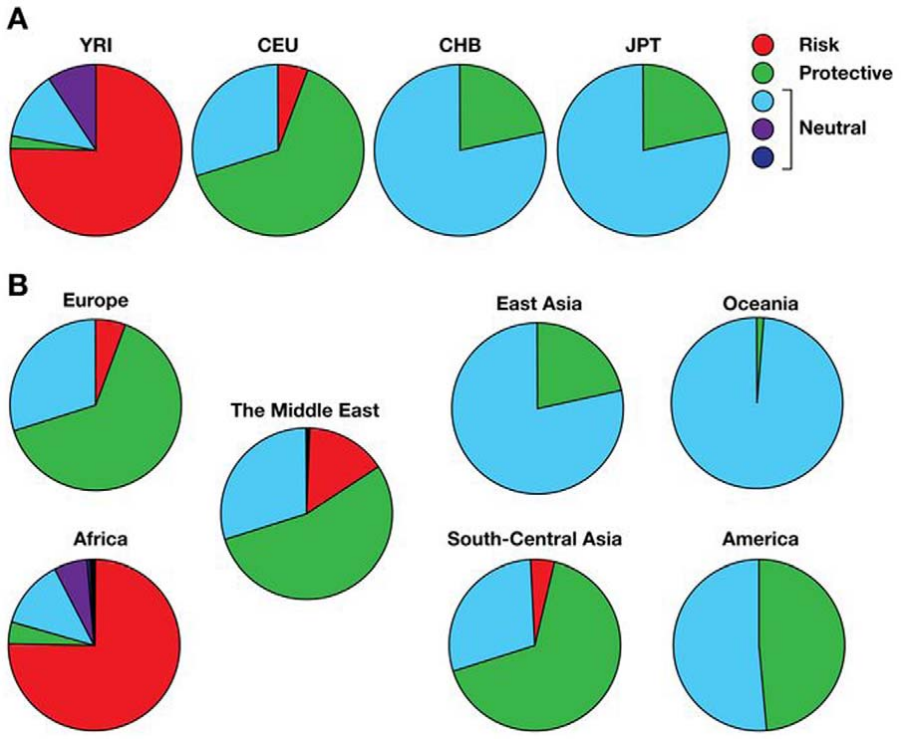
Frequencies of risk (E-1), protective (E-2), and neutral *MYH9* haplotypes (E-3-E-5) in the HapMap and HGDP. **A. Frequencies of risk (E-1), protective (E-2), and neutral haplotypes in the major populations from the HapMap** [**8**] **:** European (CEU), Yoruba (YRI), Japanese (JPT), and Chinese (CHB). Yoruba samples carry the largest proportion of the risk haplotype, while most of the Europeans in this study carry the protective haplotype (E-2). Asian populations have mainly by the neutral haplotype (E-3). **B. Frequencies of risk (E-1), protective (E-2), and neutral** ***MYH9*** **haplotypes (E-3-E-5) in the HGDP :** overall, and by the continental groups: African, European, Middle Eastern, South-Central Asian, East Asian, Oceanian, and American. African samples carry the largest proportion of risk haplotype, while most of the Europeans and the peoples in the Middle East in this study have the protective haplotype (E-2). A large proportion of the South-Central Asian populations also have the protective haplotype, while the risk haplotype is present at very low levels. East Asian and Oceanian populations are represented mainly by the neutral haplotype (E-3 and some protective haplotypes, but with the risk haplotype virtually absent.

As shown in Figure 1, the E-1 risk haplotype is prominent in Sub-Saharan Africa, especially in Yoruba, while the majority of individuals from Europe and the Middle Eastern populations feature the E-2 haplotype (Figure 1) previously reported to be protective against kidney disease (Kopp et al. 2008). The same shift can be observed in the major populations from the International HapMap Project (Figure 2) as well as in the HGDP (Figure 1). The E-2 haplotype remains frequent in South and Central Asia, but the populations in South and East Asia as well as Oceania are dominated by neutral haplotypes (E-3). In Amerindian populations, especially in Central America, the E-2 haplotype has frequencies similar to those in South and Central Asia (Figure 1). There is a decreasing north/south cline in eastern and central Asia, with higher frequencies of the protective alleles in the north compared to the south, and this may explain in part the relatively high frequencies of the protective alleles among Amerindian populations who derive from north Asian populations. The risk E-1 haplotype decreases in frequency in a cline away from Africa and is apparently extinct in East Asia, Oceania, and the Americas. It is the most common haplotype in sub-Saharan Africa; for example, estimates from HapMap or the HGDS panel indicate E-1 haplotype frequencies in the range of 69 to 80%, where the highest value is found in Yoruba. However, the prevalence of E-1 among populations in Africa is not uniformly high, with the lowest prevalence in the Mbuti and Sun populations (50%–64%). We also found substantial differentiation in frequencies of risk and protective haplotypes among human populations, as indicated by the elevated F_ST_ values between Africa and the rest of the world (F_ST_ = 0.27–0.4, Table 3 and Figure 1).

**Figure 3:**
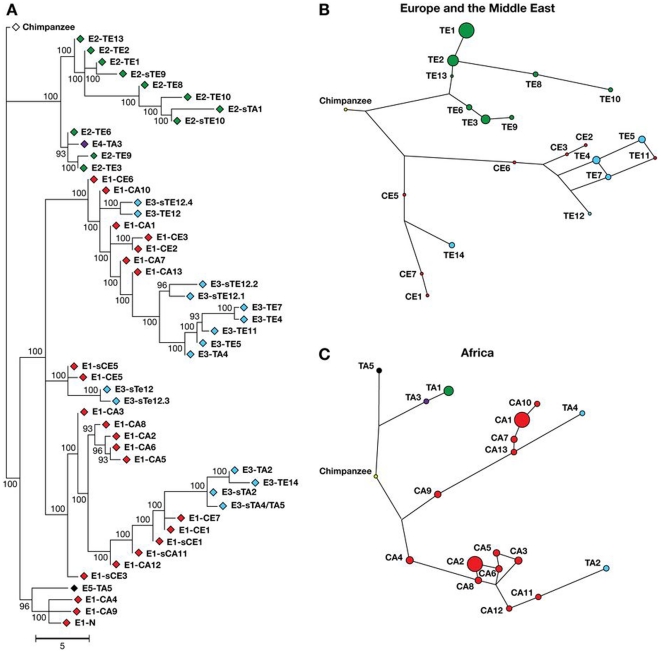
Evolutionary relationships between the extended *MYH9* haplotypes. **A.** Phylogenetic tree showing relationships between all known 27 SNP haplotypes from a combined HGDP and HapMap datasets. The colors of the nodes indicate the 4-SNP E1-E5 haplotypes and shown in the same colors as in Figure 1. Each haplotype has a notation that starts with the name of E1-E5 haplotype and extends to include the name of the extended haplotype consisting of 26 SNP and encompassing the entire *MYH9* gene. Letter codes indicate the allele at rs4821481 locus (C or T), and the origin of the reconstructed haplotype (E- Europe or Middle East; A - Africa), and the numbers are decreasing from most frequent to the least frequent in that continental population (ex.: E1-CA1 is an E-1 risk haplotype with allele C at rs4821481 locus reconstructed in Africa). The sequence and the frequencies of the common extended haplotypes (>1%) in seven continental populations from HGDP are given in Table S4. **B, C. The median joining haplotype networks representing relationships in (B) Europe and the Middle East and (C) Africa.** The networks (B and C, right) are placed opposite to the corresponding branches on the parsimony tree (A, left). The relative frequency of the particular haplotype in a population is shown by the size of the corresponding circle. Some of the haplotypes on the tree (A) are missing in the networks (B, C) due to their low frequency (<1%).

To determine the phylogenetic history of the *MYH9*, we used the HGDP genotype data to infer haplotypes for 26 SNPs between the transcription initiation and transcription termination site of the *MYH9* gene and individually matched them to the 3-SNP E block haplotypes (see Materials and Methods). The phylogenetic history of these extended haplotypes was examined using a haplotype network [20], since network approaches account for the persistence of ancestral haplotypes, the existence of multiple descendant haplotypes, recombination, and low levels of sequence variation [21]. At the same time, we also constructed a parsimony tree in MEGA 4.0.2 [22], [23] to provide an overall reference of similarity between extended haplotypes matched to the risk categories. The tree and two major continental haplotype networks (Europe+Middle East and Africa) are shown side by side in Figure 3.

The shapes of phylogenetic trees and networks suggest that the protective E-2 haplotypes (Figure 3 and Table S4, green) originated only once: they group on a single branch of the tree. This branch is scarcely represented in Africa, but diversified outside of Africa, especially in Europe and the Middle East. On the other hand, the majority of neutral E-3 haplotypes are found in Asia (Table S4), where they are often more common than E-2. However, E-3 haplotypes never cluster together (Figure 3.A and Table S4, blue): they occur on several terminal branches, always distal from the basal E-1 haplotypes (Figure 3.A and Table S4, red). This is a likely consequence of past recombination between different E-1 sequences. If risk E-1 haplotypes have an adaptive advantage in certain Sub-Saharan populations, recombination could be a quick and efficient way to provide novel and neutral genetic variation to replace previously selected sequences in populations moving to new environments. Since E-1 carries or is linked to one or more causal disease allele responsible for renal disease, when its benefits are lost outside of Africa, neutral variants generated by recombination would rise in frequency replacing the costly haplotype, as may be the case in Asian populations. This would be a likely explanation for the existence of two recombination hotspots within the gene described earlier [1].

**Figure 4:**
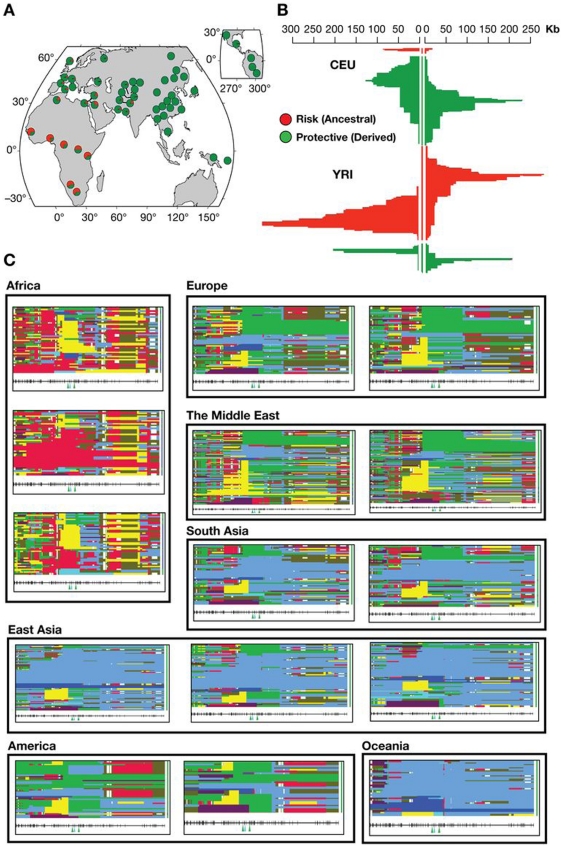
Summary of selection signatures for *MYH9*. **A. Global allele frequencies of rs4821481, an E-1 determining SNP.** The frequency of this SNP is very high in Africa resulting in an extreme F_ST_ value. This allele alone can represent risk haplotypes, since the C allele is present in over 99% of the risk (E-1) haplotype (Table 2). **B. Signature of selection in the extended haplotypes around the rs4821481 locus observed in HapMap populations (Voight et al. 2006).** The risk haplotypes are represented in red and the protective haplotypes are in green. In Yoruba, positive iHs score (iHs = 2.67) indicates that haplotypes based on the ancestral allele are longer compared to derived allele background; iHs ≥2 are considered a signature of positive selection (Voight et al. 2006). The negative values of iHs (iHS = −1.48) indicates that European haplotypes are more likely to carry the derived allele (T) for rs4821481, but the data does not provide sufficient evidence for selection on this allele. **C. Representation of extended haplotype pattern for 300 kb neighborhood around rs4821481 in the HGDP selection browser** [**11**] **.** Each box represents a single population, and observed haplotypes are shown as horizontal bars. Identical haplotypes have the same color in all of the graphs, and all haplotypes start from one of the rs4821481 alleles. To summarize the data, most continents are represented by two populations: Europe (Russian and Basque), The Middle East (Bedouin and Palestinian), South Asia (Hazara and Singhi), America (Maya and Pima). Africa (Mandenka, Yoruba, and Biaka), and East Easia (Han, Yakut and Japanese) are represented by three populations each, and Oceania is represented by a single population (Papuan). An unusual pattern in Africa is confined to the Yoruba population (see Figure 4.B). In the West (Europe, Middle East) versus the East Eurasia (East and South Central Asia), different haplotypes predominate concordantly with our earlier conclusions (see the worldwide map of risk haplotypes in Figure 1).

The pattern of LD across *MYH9* (Figure S1) reflects a general decrease in diversity with African populations exhibiting the most haplotype diversity, intermediate levels in Europe and Asia, and the least in Oceania and the Americas. The gray areas in the LD plots, most extensive in the African groups, represent SNP comparisons where haplotypes could not be inferred robustly due to high recombination rates. The evolutionary history of *MYH9* is complex. Several recent whole-genome scans indicate that *MYH9* has been the subject of natural selection during different periods of human evolution, starting from the time of human-primate divergence, and extending to the more recent local adaptations in modern populations. *MYH9* exhibits a paucity of amino acid divergence between humans and *Pan troglodytes,* yet human *MYH9* has moderate to high levels of amino acid polymorphism. Bustamante et al., [24] suggests that this may indicate an excess of mildly deleterious variation possibly due to balancing selection. Among Yoruba samples included in Hapmap, haplotypes centered on rs4821481 can be observed in the Happlotter browser [13] to exhibit extended haplotype homozygosity compared to the protective allele (iHs = 2.67), indicating a possible selection signature (Figure 4.B, red). The same can be observed in the HGDP selection browser: in Yoruba, the pattern of extended haplotype homozygosity is markedly different from that in other African populations such as Mandenka and Biaka, also from west Africa (Figure 4.C). The most frequent extended haplotype in this population in CA2 (Figure 2A and C, Table S4) indicating that this haplotype is under selection. Other related haplotypes may also be involved (CA3, CA5, CA6 and CA8, Figure 2.C, Table S4). If confirmed, such evolutionary scenario for the *MYH9* region will be reminiscent of that described for the candidate genes in the thrifty gene hypothesis where the ‘thrifty’ genotype would have been historically advantageous for the local population, because it offered protection from disease, or some other environmental factor, while in the modern times, when the protective effect is no longer needed, phenotypic effects persist as health conditions [25], [26].

The combination of high frequency extended haplotypes and high F_ST_ values is a possible signal of recent positive selection for the kidney risk haplotype in the Yoruba. This raises the possibility that the *MYH9* risk haplotype in Africa has hitchhiked along with a different allele in *MYH9* or in one of the neighboring genes, such as *APOL1*, encoding apolipoprotein L-I, located only 14 kb away on chromosome 22, at 34,979,070–34,993,484. Since *APOL1* is involved in the resistance to infection by *Trypanosoma brucei*, the cause of African trypanosomiasis or sleeping sickness in sub-Saharan Africa [27], [28], there is a tantalizing possibility that a *MYH9* risk haplotype in Africa has risen in frequency hitchhiking on the selective sweep of the nearby *APOL1* gene. Specifically, we suggest that there may be a heterozygote advantage to carrying a single risk chromosome that confers protection against a pathogen such as malaria or sleeping sickness, and a homozygous disadvantage that predisposes to kidney disease in carriers of a single risk allele. This possibility can be addressed by the future studies involving extended haplotypes around the resistance locus in *APOL1* in the relevant populations in West Africa.

There is also a possibility that *MYH9* has been selected by *Plasmodium falciparum*, since *MYH9* is a negative regulator of platelet biogenesis [29], and platelets are involved in cerebral malaria and pathogenesis. The Yoruba *MYH9* haplotypes seem to be unique among other African populations, since the extent of haplotype homozygosity in this population is greater than in the neighboring populations from Africa (Figure 4.C)[11]. In the European population the derived allele (T) tagging the protective haplotypes features extended LD with the neighboring haplotypes relative to the alternative allele, but the difference is too small to suggest recent selection of the E-2 haplotype (iHs = −1.48, Figure 4.B, in green)[13]. This, however, does not rule out the presence of selection signature in the recent past, since iHs is sensitive to the occurrence of multiple equally long adaptive haplotypes, or a more ancient one that may be detected by other methods [12]. Importantly, while E-1 haplotype is a good approximation, the causative SNP for the renal disease has not been identified [1], [2]. The extended LD in the *MYH9* region and the extended haplotype homozygosity noted in Yoruba confound the identity of the true causal variation predisposing to increased risk of kidney disease identified in African Americans who share ancestry with west Africans represented here by the Yoruba [30].

The public health impact of *MYH9* risk haplotypes in sub-Saharan Africa may be considerable, given the substantial burden of HIV-1 disease throughout Africa and the high frequency of *MYH9* risk alleles in African populations. Surveys of HIV-associated kidney disease and chronic kidney disease across sub-Saharan are sporadic and inconsistent in diagnostic criteria. However recent estimates of chronic kidney disease among individuals with HIV-1 disease range from an estimated prevalence of 6% in South Africa blacks, 38% in Nigeria, and 48% in Uganda [31]. Notably, HIVAN is not reported in East African Ethiopian Jews with African ancestry [32]. Chronic kidney disease in the general population is not well studies however, estimates from the Democratic Republic of Congo report CKD report rates of 12.4% for all stages of chronic kidney disease diagnosed by decreased estimated glomerular filtration rates (<60 ml/min/1.73 m) or proteinuria (≥300 mg/day) with a high prevalence of proteinuria not due to hypertension or diabetes consistent with glomerulopathies such as FSGS or HIVAN [33]. South Africa also reports high rates of chronic kidney disease; hypertension effects 25% of the adult black population and is the cause of kidney failure in 21% of patients on dialysis [31].

HIVAN is the third leading cause of ESKD in African American men between the ages of 20–64 years [34] and end stage renal disease is 4 times more frequent in the African American population. In African Americans ESKD attributed to hypertension is strongly associated with *MYH9* region risk alleles [1]–[3]. The present study provides insights into the global distribution of *MYH9* risk alleles and haplotypes and may be useful in forming public health policies to mitigate and reduce the added burden of kidney disease in vulnerable populations.

In conclusion, our results suggest that the *MYH9* risk alleles and haplotypes are notably differentiated among human populations that can be attributed to the interplay of geographic, demographic and evolutionary factors, leading to striking differences between African and non-African populations in genetic risk for chronic kidney disease. More research is needed to understand which factors account for these population differences. Understanding haplotype structure, evolutionary history and the role of natural selection in the MYH9 region are crucial next steps that may reveal the true causal renal susceptibility loci.

## Materials and Methods

### Ethics Statement

Institutional review boards at National Cancer Institute and National Institute of Diabetes and Digestive and Kidney Diseases, National Institutes of Health, approved the study protocols.

### Data and Samples Studied

We obtained genotypes from the two primary sources of genome-wide SNPs: the CEPH Human Genome Diversity Panel (HGDP) and the International HapMap Project (Phase II). These two datasets provide the best geographic sampling currently available [8], [9]. The CEPH-HGDP genotype data consists of 640,000 SNPs for 938 individuals representing 51 global populations reported by Li et al [9]. Phase II of the International HapMap Project genotyped 210 individuals for over 3 million SNPs from four populations: Yoruba from Ibadan, Nigeria (YRI); Chinese Han from Beijing, China (CHB); Japanese from Tokyo, Japan (JPT); and Utah residents with ancestry from northern and western Europe (CEU) [8]. A single SNP, rs4821481 in the E haplotype block was previously genotyped on the HGDP panel and available for download [9].

### Genotyping and Haplotype Reconstruction

TaqMan assays (Applied Biosystems, Foster City CA) were used to genotype HGDP DNA samples from the Foundation Jean Dausset-CEPH, Paris, France [9]. Genotypes were obtained for 963 individuals from 51 distinct ethnic groups for rs3752462 and rs4821480 (previously reported tagging SNPs). E block haplotypes were inferred from three of the four defining SNPs, omitting rs2032487; this allowed inferring haplotypes E-1, E-2, E-4, and E-5 with nearly 100% accuracy and haplotype E-3 with 95% accuracy. To provide additional information for haplotype reconstruction and increase the accuracy of inference, SNPs rs2157257, rs5750250, and rs3830104 were also genotyped.

Since there were missing genotypes for rs4821481 (54%), we used neighboring SNPs (rs2157257, rs5750250, and rs3830104) known to be in strong LD with the missing marker to impute the haplotypes for these samples. Haplotypes were reconstructed using combined data within each population separately by implementation of the expectation-maximization (EM) algorithm available in SAS/Genetics package (SAS 9.1.3, Cary NC). In populations with many missing genotypes, uncertainty of the estimates increased, so more than one haplotype was assigned to the same chromosome. To compensate for this ambiguity, all possible haplotype pairs in each individual were weighted by their posterior probabilities [35], then partial probabilities for each haplotype were accounted for; the inferred haplotype frequencies in each population reflect uncertainties of the estimates.

### Data Analysis

Allele and genotype frequencies, and observed heterozygosities for alleles and haplotypes were estimated using SAS Genetics (SAS 9.1.3, Cary NC). F_ST_ was used to assess population divergence: high F_ST_ indicates that most of the variance in allele frequencies comes from the difference between populations used in the comparison [36]–[37]. Under neutral conditions, F_ST_ is determined by genetic drift affecting all loci across the genome in a similar way, but selection can cause differences between populations in the locus and the surrounding genomic region [1], [18], [19], [38]. For inferred haplotypes, we calculated pairwise conventional F_ST_ and carried out 10,000 permutations for significance using Arlequin 3.1 [39]. Pairwise F_ST_ values were calculated between continental groups, and between the individual populations based on the continental groupings identified by Rosenberg et al. [10]. We also used F_ST_ values from HGDP selection browser [11] and Happlotter [13], since these studies used essentially the same datasets (HGDP and HapMap, respectively).

Since a subset of HGDP samples have been genotyped for 26 SNPs covering the entire span of *MYH9* [9], [40], [41] on the same samples we were analyzing, we were able to match these extended haplotypes to the five E haplotype risk categories of *MYH9* for renal disease (E-1 to E-5). The phylogenetic history of the extended haplotypes was examined using a haplotype network determined by the Network 4.5 program [20]. Network approaches have several advantages over traditional phylogenetic methods such as trees, since they account for the persistence of ancestral haplotypes, the existence of multiple descendant haplotypes, recombination and low levels of sequence variation [21]. The networks were constructed by first connecting haplotypes that differed by single nucleotide changes and next adding increasingly more distant haplotypes. The process was carried on until either all available haplotypes were included, or the maximum number of mutational steps was reached.

Since we used 26 SNPs to infer risk and protective haplotypes, each of the reported risk (E-1), protective (E-2) and neutral haplotypes (E-3–5) include a number of haplotypes defined by additional SNPs (two SNPS genotyped by us originally and defining E-1-E-5, rs4821481 and rs3830104 [1], plus 24 additional SNPs). To provide an overall reference of similarity between extended haplotypes matched to the risk categories, a parsimony tree was constructed using MEGA 4.0.2 [22], [23]. The phylogeny was inferred using the Maximum Parsimony method [42]. The consensus tree inferred from 30 most parsimonious trees was displayed, and the percentage of parsimonious trees in which the associated taxa clustered together are shown next to the branches (Figure 2).

To assess the possibility of a selection signature surround the E haplotypes, we utilized the Integrated Haplotype Score (iHs) values from the Haplotter [13] and HGDP selection browser [11]. This measure has been developed to detect evidence of recent positive selection at a locus and considers differential levels of linkage disequilibrium (LD) surrounding a putatively selected allele by comparing it to LD around the alternative allele at the same position [13]. The iHS statistic is related to the Extended Haplotype Homozygosity test [43], but is considered to have more power to detect selection with sweeps that reach intermediate frequencies, rather than complete sweeps leading to fixation [44]. A positive iHs score (iHs ≥ 2) indicates that haplotypes based on the ancestral allele are longer compared to those extending from the derived allele, while negative values of the iHs statistics (iHs ≤−2) indicate that the derived allele is under selection [13].

## Supporting Information

Table S1 Pairwise Fst between African populations from HGDP and HapMap.
(0.04 MB DOC)

Table S2 Pairwise Fst between European populations from HGDP.
(0.04 MB DOC)

Table S3 Pairwise Fst between the Middle Eastern populations from HGDP.
(0.03 MB DOC)

Table S4 Representation of extended haplotypes of *MYH9* (26 SNP) in seven continental populations from HGDP. The frequency is given as a percentage of the total individuals in a population and colored so the darker is the cell, the more common is the haplotype. Rare haplotypes (<1%) are not shown. The data was obtained from Pemberton et al. [40].
(0.14 MB DOC)

Figure S1 Linkage disequilibrium heat plots for 51 Human Diversity Panel ethnic groups, showing D' for 44 *MYH9* SNPs extending from rs2012928 to rs738278, encompassing *MYH9* and about 10 Kb on either side. LD was calculated from haplotype frequencies; haplotypes were estimated using the EM method. Haplotype inference was carried out to a length of 14 SNPs, hence the bottom of the charts is gray (no inference). Gray squares closer to the top of the chart indicate regions where haplotypes could not be reliably inferred due to extreme LD. Notably, LD is much greater in the African groups than other continental groups; diversity is minimum for the Americas and Oceania.
(3.31 MB PDF)
