# Supplementary material for: Worldwide Distribution of the MYH9 Kidney Disease Susceptibility Alleles and Haplotypes: Evidence of Historical Selection in Africa
Source: PLoS One. 2010 Jul 9;5(7):e11474. doi: 10.1371/journal.pone.0011474 (PMC2901326; doi:10.1371/journal.pone.0011474)
Supplement: Table S1 — Pairwise Fst between African populations from HGDP and HapMap. (0.04 MB DOC) [file pone.0011474.s001.doc]

**Table S1.** **Pairwise F_ST_ between African populations from HGDP and HapMap**

| # | Population | 1 | 2 | 3 | 4 | 5 | 6 | 7 | 8 | 9 |
| --- | --- | --- | --- | --- | --- | --- | --- | --- | --- | --- |
| 1 | AFRICA* |  | - | + | - | - | - | - | - | + |
| 2 | BANTU-KENYA | -0.012 |  | + | - | - | - | - | - | - |
| 3 | BANTU-SA | **0.300** | **0.192** |  | + | + | - | - | + | + |
| 4 | BIAKA | 0.009 | -0.013 | **0.239** |  | - | + | - | - | + |
| 5 | MANDENKA | -0.010 | -0.033 | **0.215** | -0.025 |  | - | - | - | + |
| 6 | MBUTI | 0.132 | 0.014 | 0.032 | **0.079** | 0.065 |  | - | + | + |
| 7 | SAN | 0.170 | 0.069 | 0.074 | 0.058 | 0.083 | -0.016 |  | + | + |
| 8 | YORUBA | -0.008 | 0.013 | **0.373** | 0.031 | 0.009 | **0.186** | **0.240** |  | - |
| 9 | YRI** | **0.015** | 0.050 | **0.440** | **0.075** | **0.047** | **0.255** | **0.325** | -0.022 |  |

* Comparison of each population to the combined sample including all individuals from the continent.

** HapMap sample of Yoruba
