# Supplementary material for: Worldwide Distribution of the MYH9 Kidney Disease Susceptibility Alleles and Haplotypes: Evidence of Historical Selection in Africa
Source: PLoS One. 2010 Jul 9;5(7):e11474. doi: 10.1371/journal.pone.0011474 (PMC2901326; doi:10.1371/journal.pone.0011474)
Supplement: Table S2 — Pairwise Fst between European populations from HGDP. (0.04 MB DOC) [file pone.0011474.s002.doc]

**Table S2.** Pairwise F_ST_-s between European populations from HGDP

| # | Population | 1 | 2 | 3 | 4 | 5 | 6 | 7 | 8 | 9 |
| --- | --- | --- | --- | --- | --- | --- | --- | --- | --- | --- |
| 1 | ADYGHEI |  | - | - | - | - | - | - | - | - |
| 2 | BASQUE | -0.003 |  | - | - | - | - | - | - | - |
| 3 | CEU *** | -0.004 | -0.023 |  | - | - | - | - | - | - |
| 4 | FRENCH | -0.019 | -0.030 | -0.022 |  | - | - | - | - | - |
| 5 | ITALIAN | -0.053 | -0.030 | -0.028 | -0.041 |  | - | - | - | - |
| 6 | ORCADIAN | 0.005 | -0.040 | -0.016 | -0.017 | -0.020 |  | - | - | - |
| 7 | RUSSIAN | -0.015 | -0.032 | -0.019 | -0.025 | -0.039 | -0.046 |  | - | - |
| 8 | SARDINIAN | -0.021 | -0.030 | -0.021 | -0.031 | -0.050 | -0.022 | -0.027 |  | - |
| 9 | TUSCAN | -0.033 | -0.072 | -0.056 | -0.072 | -0.051 | -0.057 | -0.057 | -0.058 |  |

*** HapMap sample of CEU
