# Supplementary material for: Worldwide Distribution of the MYH9 Kidney Disease Susceptibility Alleles and Haplotypes: Evidence of Historical Selection in Africa
Source: PLoS One. 2010 Jul 9;5(7):e11474. doi: 10.1371/journal.pone.0011474 (PMC2901326; doi:10.1371/journal.pone.0011474)
Supplement: Table S3 — Pairwise Fst between the Middle Eastern populations from HGDP. (0.03 MB DOC) [file pone.0011474.s003.doc]

**Table S3.** Pairwise F_ST_ between the Middle Eastern populations from HGDP

| # | Population | 1 | 2 | 3 | 4 |
| --- | --- | --- | --- | --- | --- |
| 1 | BEDOUIN |  | - | - | - |
| 2 | DRUZE | 0.024 |  | - | - |
| 3 | MOZABITE | 0.003 | -0.003 |  | - |
| 4 | PALESTINIAN | 0.013 | -0.013 | -0.012 |  |
