# Supplementary material for: Worldwide Distribution of the MYH9 Kidney Disease Susceptibility Alleles and Haplotypes: Evidence of Historical Selection in Africa
Source: PLoS One. 2010 Jul 9;5(7):e11474. doi: 10.1371/journal.pone.0011474 (PMC2901326; doi:10.1371/journal.pone.0011474)
Supplement: Table S4 — Representation of extended haplotypes of MYH9 (26 SNP) in seven continental populations from HGDP. The frequency is given as a percentage of the total individuals in a population and colored so the darker is the cell, the more common is the haplotype. Rare haplotypes (<1%) are not shown. The data was obtained from Pemberton et al. [40]. (0.14 MB DOC) [file pone.0011474.s004.doc]

**Table S4. Representation of extended haplotypes of *MYH9* (26 SNP) in seven continental populations from the HGDP.**

The frequency is given as a percentage of the total individuals in a population and colored so the darker is the cell, the more common is the haplotype.

Rare haplotypes (<1%) are not shown. The data was obtained from Pemberton et al. [40].

|  | **Risk Haplotype** | **Haplotype** | **AFRICA** | **M_EAST** | **EUROPE** | **CS_ASIA** | **E_ASIA** | **OCEANIA** | **AMERICA** | **WORLD** | **Sequence†** |
| --- | --- | --- | --- | --- | --- | --- | --- | --- | --- | --- | --- |
|  | **Ancestral alleles** |  |  |  |  |  |  |  |  |  | **AGNNGCCCGGTAATCGCCCCGGTAAC** |
|  | E-1 | sCE5 |  |  | 1 | 1 |  |  |  |  | **AATTGCCCGATGATCGCCCCGGTGAC** |
|  | E-1 | sCE3 | 1 |  |  |  |  |  |  |  | **AATTGCCCGATGACTACCCCGTTAAC** |
|  | E-1 | sCE1 | 1 |  |  |  |  |  |  |  | **CGCTGCCCGATGACTACCCCGGTGAC** |
|  | E-1 | CE7 |  | 1 | 1 |  |  |  |  |  | **CGCTGCCCGATGACCACCCCGGTGAA** |
|  | E-1 | CE6 |  | 1 | 1 |  |  |  |  |  | **AATTGCCCGATGATCGTACCGTCAGC** |
|  | E-1 | CE5 |  |  | 2 |  |  |  |  |  | **AATTGCCCGATGATCGCCCCGGTGAA** |
|  | E-1 | CE3 |  | 2 |  |  |  |  |  | 1 | **AATTGCCCGATGATCATCCCATCAGC** |
|  | E-1 | CE2 | 1 | 2 |  |  |  |  |  | 1 | **AATTGCCCGATGATTATCCCATCAGC** |
|  | E-1 | CE1 |  | 5 |  | 1 |  |  |  | 1 | **CGCTGCCCGATGACCACCCCGGTGAC** |
|  | E-1 | CA9 | 2 |  |  |  |  |  |  |  | **CGTTGCCCGGTAATTGTCCCGTTAGC** |
|  | E-1 | CA8 | 2 |  |  |  |  |  |  |  | **CGTTGCCCGATGATTACCTCGTTAAC** |
|  | E-1 | CA7 | 2 |  |  |  |  |  |  | 1 | **AGTTGCCCGATGATTGTACCATCAGC** |
|  | E-1 | CA6 | 2 |  |  |  |  |  |  | 1 | **AGTTGCCCGATGATTACCTCGTTAAA** |
|  | E-1 | CA5 | 2 | 1 |  |  |  |  |  | 1 | **AATTGCCCGATGATTACCTCGTTAAA** |
|  | E-1 | CA4 | 3 |  |  |  |  |  |  | 1 | **CGTTGCCCGGTAATCATCTCGTTAAC** |
|  | E-1 | CA3 | 3 |  |  |  |  |  |  | 1 | **AATTGCCCGATGATTACCTCGTTAAC** |
|  | E-1 | CA2 | 12 |  |  |  |  |  |  | 3 | **CGTTGCCCGATGATTACCTCGTTAAA** |
|  | E-1 | CA13 | 2 |  |  |  |  |  |  |  | **CGTTGCCCGATGATTGTACCATCAGC** |
|  | E-1 | CA12 | 2 |  |  |  |  |  |  |  | **AGTTGCCCGATGATTACCTCGGTGAC** |
|  | E-1 | CA11 | 2 |  |  |  |  |  |  |  | **AGTTGCCCGATGACTACCCCGGTGAC** |
|  | E-1 | CA10 | 2 |  |  |  |  |  |  |  | **AATTGCCCGATGATCGTACCATCAGC** |
|  | E-1 | CA1 | 14 |  |  | 1 |  |  |  | 3 | **AATTGCCCGATGATTGTACCATCAGC** |
|  | E-2 | TE9/TA1 | 5 | 3 | 1 | 2 | 1 |  |  | 2 | **AATCATCCAGTAGTCGCCCCGGTGAA** |
|  | E-2 | TE8 |  | 2 | 7 |  |  |  |  | 1 | **CGCTATCCAGTAGTCGTACCAGCGGC** |
|  | E-2 | TE6 |  | 5 | 5 |  |  |  |  | 2 | **AATTATCCAGTAGTCGCCCCGGTGAC** |
|  | E-2 | TE3 |  | 8 | 7 | 4 | 1 |  | 1 | 4 | **AATTATCCAGTAGTCGCCCCGGTGAA** |
|  | E-2 | TE2 | 1 | 11 | 12 | 21 | 4 |  | 14 | 9 | **CGCTATCCAGTAGTCGCCCCGGTGAC** |
|  | E-2 | TE13 |  |  | 2 | 1 |  |  |  |  | **AGCTATCCAGTAGTCGCCCCGGTGAC** |
|  | E-2 | TE10 |  | 1 | 4 |  |  |  |  | 1 | **CGCTATCCAGTAGTCATACAATCAGC** |
|  | E-2 | TE1 | 1 | 19 | 32 | 11 | 3 |  | 15 | 13 | **CGCTATCCAGTAGTCGCCCCGGTGAA** |
|  | E-2 | sTE9 | 1 |  |  |  |  |  |  |  | **CGTCATCCAGTAGTCGCCCCGGTGAA** |
|  | E-2 | sTE10 |  |  |  | 4 | 4 |  | 3 | 1 | **CGCTATCCAGTAGCCGTACCATCAGC** |
|  | E-2 | sTA1 |  |  |  |  | 2 |  | 1 | 0 | **AATCATCCAGTAGCCGTACCATCAGC** |
|  | E-3 | TE7 |  | 4 | 2 | 2 | 1 |  |  | 2 | **AATCGTCTGATGACCGTACCATCAGA** |
|  | E-3 | TE5 |  | 5 | 6 | 14 | 53 | 69 | 29 | 17 | **CGCCGTCTGATGACCGTACCATCAGC** |
|  | E-3 | TE4 |  | 8 | 2 | 18 | 2 | 3 |  | 5 | **AATCGTCTGATGACCGTACCATCAGC** |
|  | E-3 | TE14 |  | 1 | 1 |  |  |  |  |  | **AATCGTCTGATGACCGCCCCGGTGAA** |
|  | E-3 | TE12 |  | 1 | 1 |  |  |  |  |  | **AATTGTCCGACGATCGTACCATCAGA** |
|  | E-3 | TE11 |  | 2 | 2 |  | 1 |  |  | 1 | **CGCCGTCTGATGACCGTACCATCAGA** |
|  | E-3 | TA4 | 1 |  |  |  | 1 | 6 | 4 | 1 | **CGCCGTCTGATGACTGTACCATCAGC** |
|  | E-3 | TA2 | 2 |  |  |  |  |  |  |  | **AGTCGTCTGATGACCGCCCCGGTGAC** |
|  | E-3 | sTE12 |  |  |  |  | 3 | 16 | 2 | 1 | **CGCTGTCCGACGACCGTACCATCAGC** |
|  | E-3 | sTE12 |  |  |  |  | 1 | 3 | 5 | 1 | **CGCTGTCCGACGATTATACAATCAGC** |
|  | E-3 | sTE12 |  |  |  | 1 | 2 |  |  |  | **AATTGTACGACGATCGCCCCGGTGAC** |
|  | E-3 | sTE12 |  |  |  | 1 | 1 |  |  |  | **AATTGTACGACGATCGCCCCGGTGAA** |
|  | E-3 | sTE12 |  |  |  | 1 | 1 |  |  |  | **AATTGTACGACGATCGTACCATCAGC** |
|  | E-3 | sTA4/TA5 |  | 1 |  | 1 | 1 |  | 1 |  | **CGCCGTCTGATGATCGCCCCGGTGAC** |
|  | E-3 | sTA2 |  | 1 |  | 1 | 1 |  |  |  | **CGCCGTCTGATGACCGCCCCGGTGAC** |
|  | E-4 | TA3 | 1 | 1 | 1 |  | 7 | 3 | 12 | 3 | **AATCATCCAGTAGTCGCCCCGGTGAC** |
|  | E-5 | TA5 | 1 |  |  |  |  |  |  |  | **AATCGTCCGGTAATCGTCCCGTTAGC** |

**†** The sequence of loci in the extended haplotypes is rs136187, rs136196, rs735854, rs5756129, rs875726, rs4821481, rs2239787, rs2239781,

rs1557530, rs2157256, rs3830104, rs4820230, rs8141971, rs9610489, rs2239784, rs1005570, rs6000239, rs8136069, rs16996672, rs11704382,

rs4820234, rs2413398, rs1557540, rs713839, rs739097, rs11089788. Ancestral alleles are downloaded from the NCBI page for each SNP.
