# Supplementary material for: Worldwide Distribution of the MYH9 Kidney Disease Susceptibility Alleles and Haplotypes: Evidence of Historical Selection in Africa
Source: PLoS One. 2010 Jul 9;5(7):e11474. doi: 10.1371/journal.pone.0011474 (PMC2901326; doi:10.1371/journal.pone.0011474)
Supplement: Figure S1 — Linkage disequilibrium heat plots for 51 Human Diversity Panel ethnic groups, showing D' for 44 MYH9 SNPs extending from rs2012928 to rs738278, encompassing MYH9 and about 10 Kb on either side. LD was calculated from haplotype frequencies; haplotypes were estimated using the EM method. Haplotype inference was carried out to a length of 14 SNPs, hence the bottom of the charts is gray (no inference). Gray squares closer to the top of the chart indicate regions where haplotypes could not be reliably inferred due to extreme LD. Notably, LD is much greater in the African groups than other continental groups; diversity is minimum for the Americas and Oceania. (3.31 MB PDF) [file pone.0011474.s005.pdf]

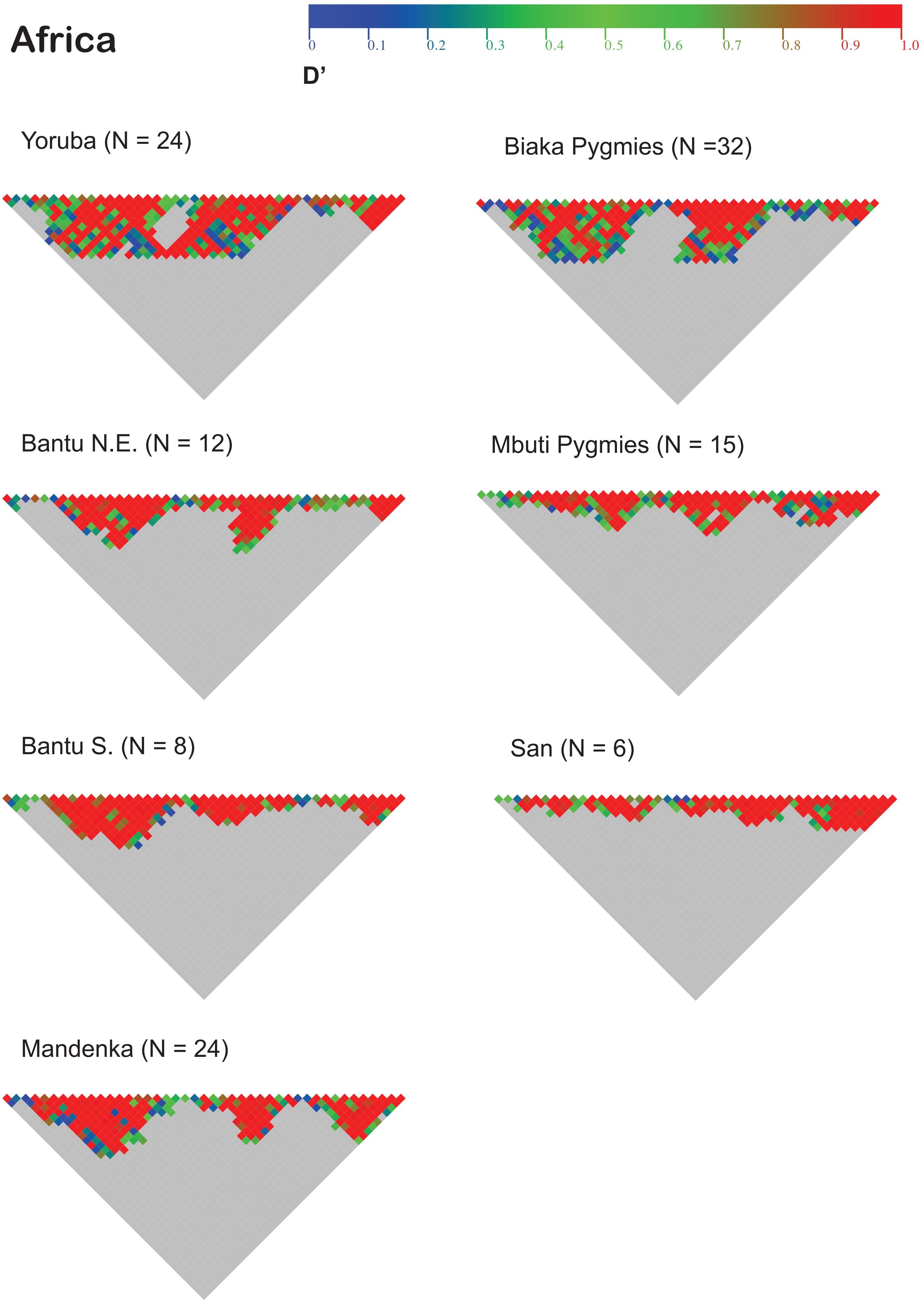
Africa
0
0.1
0.2
0.3
0.4
0.5
0.6
0.7
0.8
0.9
1.0
D'
Yoruba (N = 24)
Biaka Pygmies (N =32)
Bantu N.E. (N = 12)
Mbuti Pygmies (N = 15)
Bantu S. (N = 8)
San (N = 6)
Mandenka (N = 24)

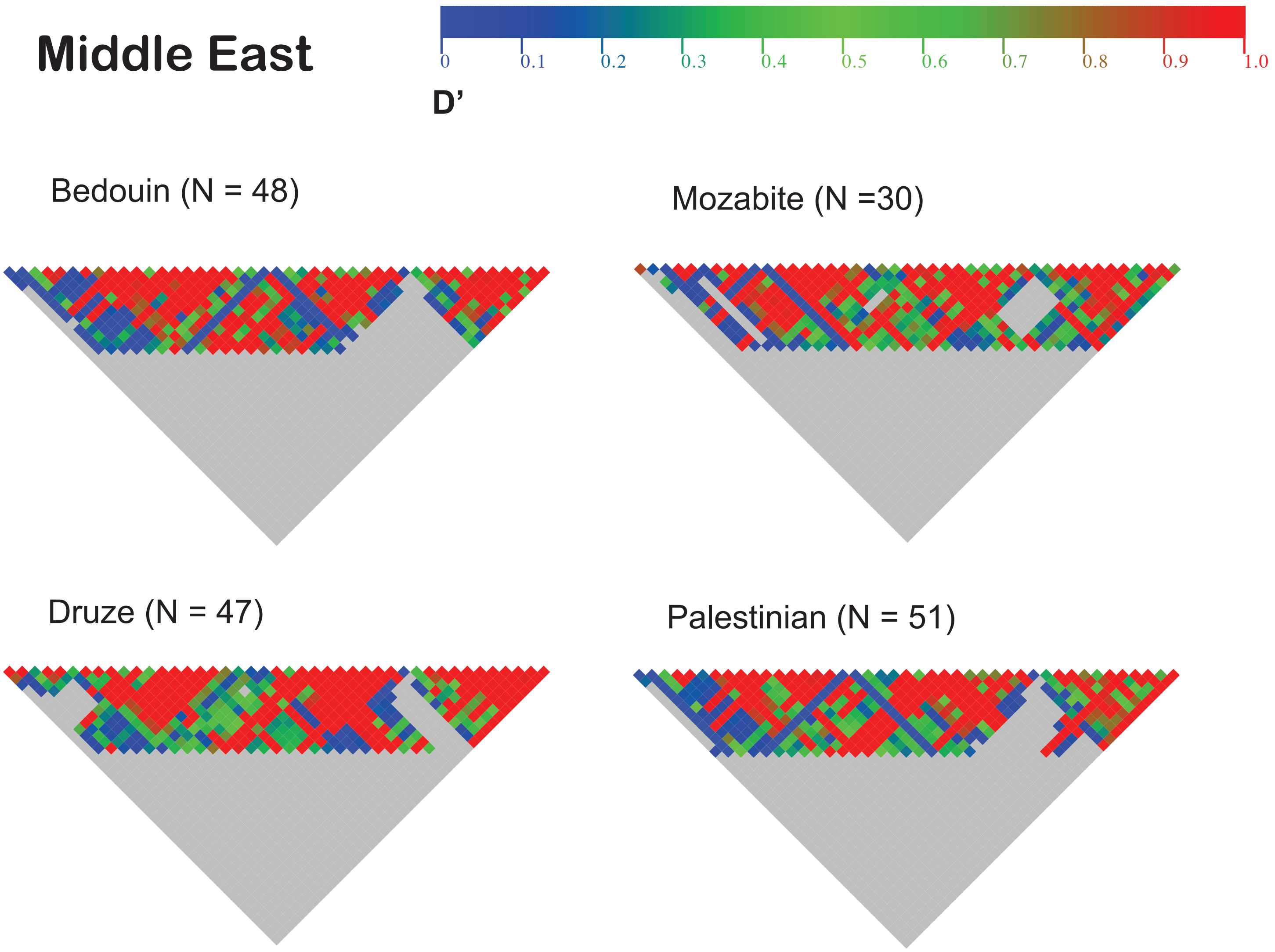
Middle East
0
0.1
0.2
0.3
0.4
0.5
0.6
0.7
0.8
0.9
1.0
D’
Bedouin (N = 48)
Mozabite (N =30)
Druze (N = 47)
Palestinian (N = 51)

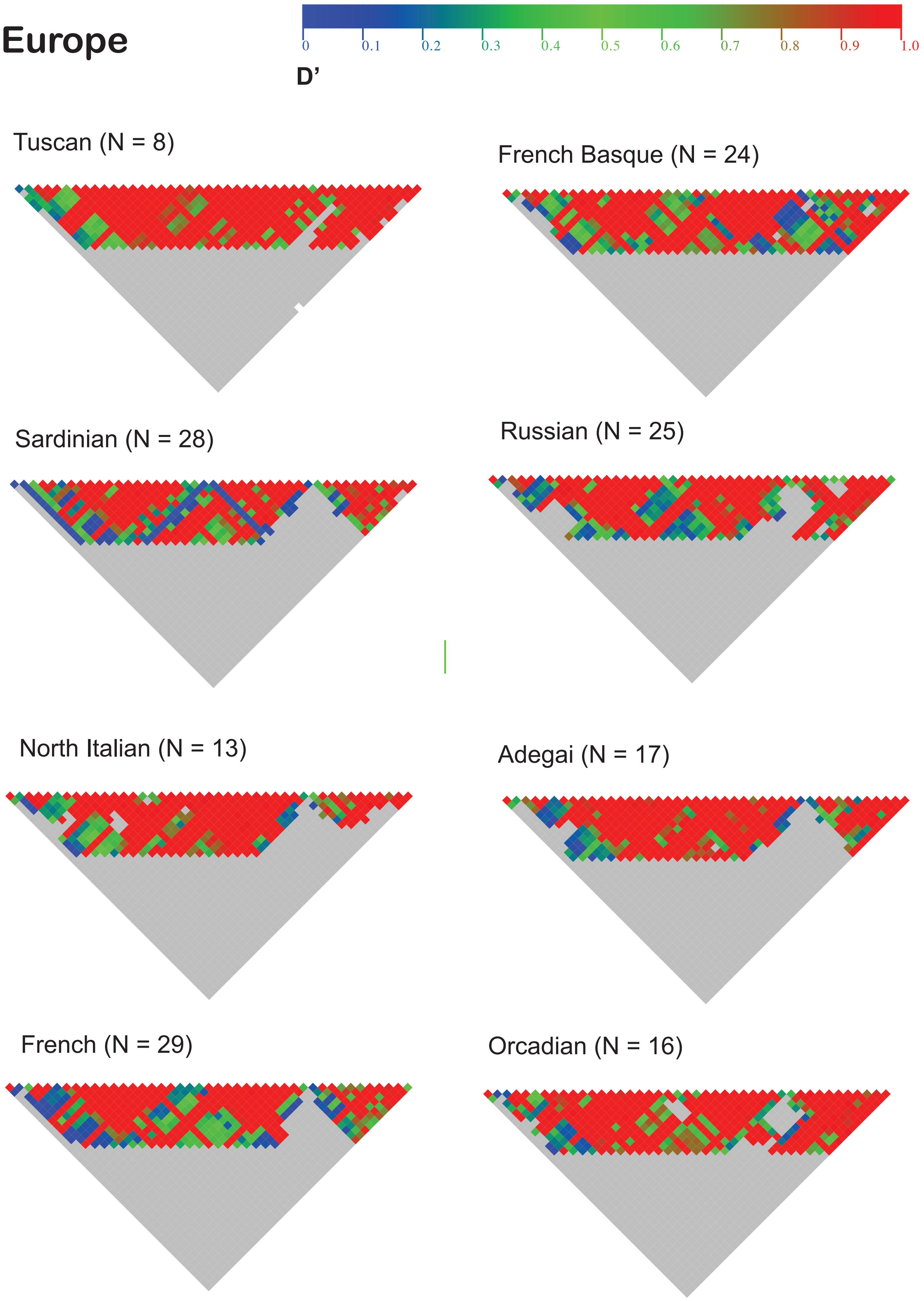
Europe
0
0.1
0.2
0.3
0.4
0.5
0.6
0.7
0.8
0.9
1.0
D’
Tuscan (N = 8)
French Basque (N = 24)
Sardinian (N = 28)
Russian (N = 25)
North Italian (N = 13)
Adegai (N = 17)
French (N = 29)
Orcadian (N = 16)

## Central/South Asia (1)

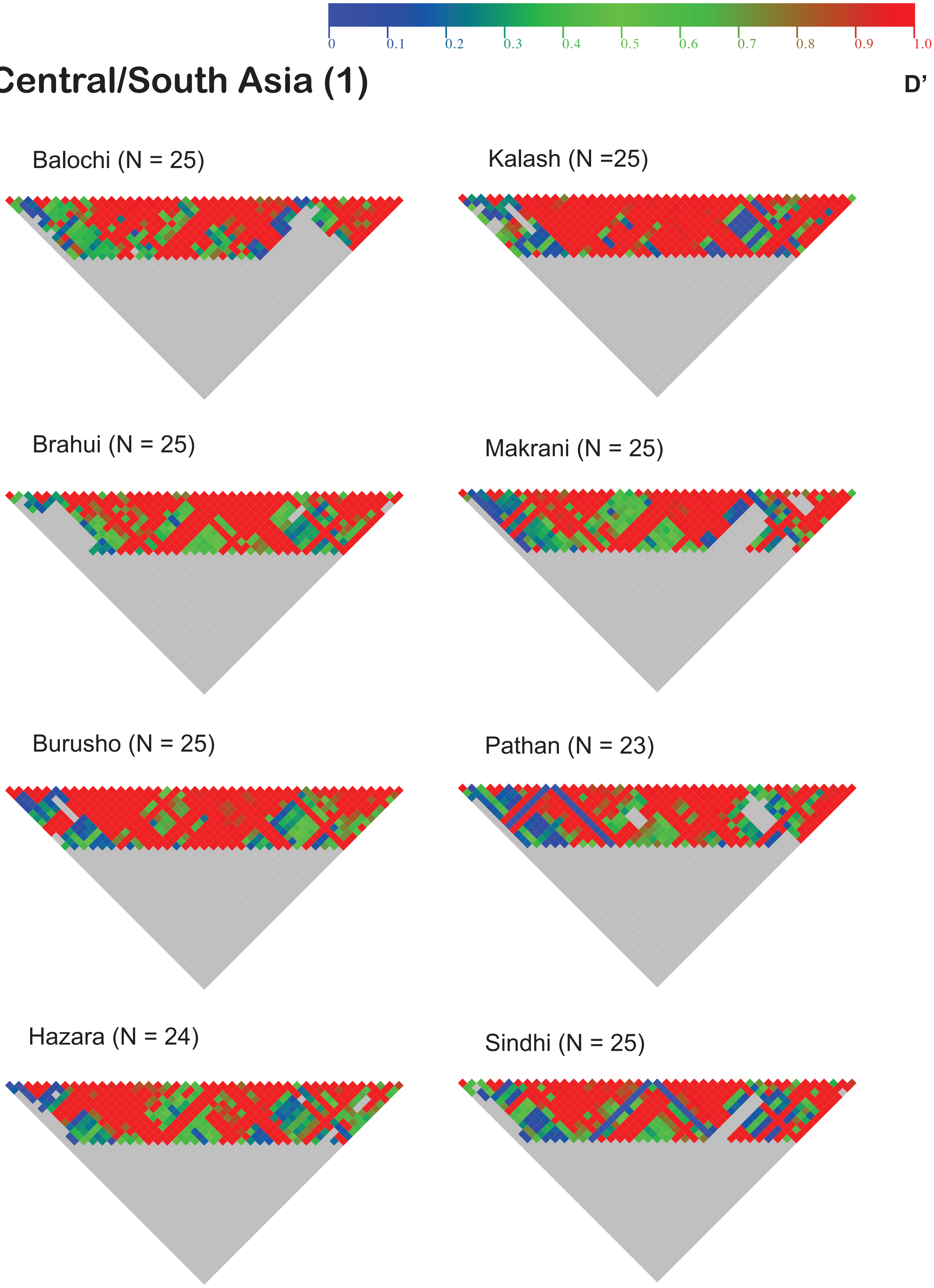

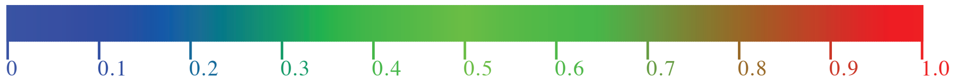


# Central/South Asia (2)

D’

Uygur (N = 10)

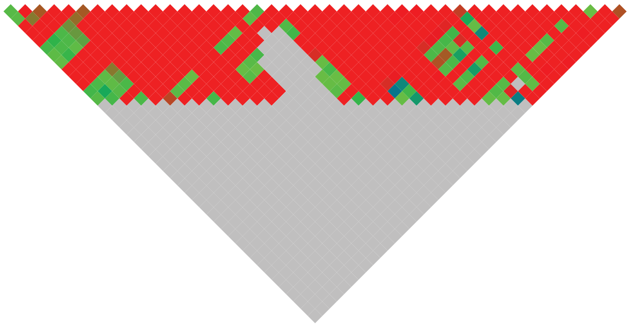

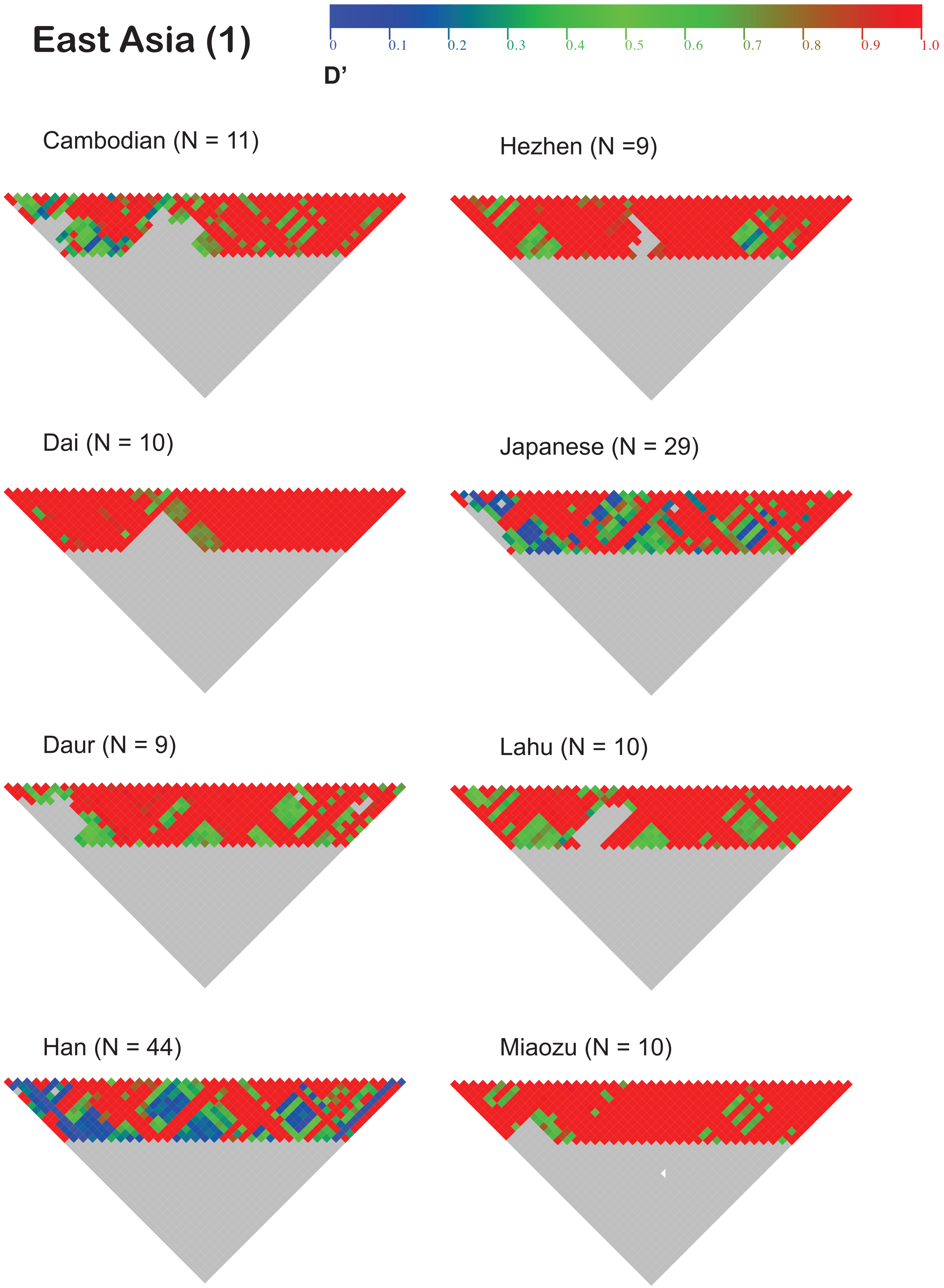
East Asia (1)
0
0.1
0.2
0.3
0.4
0.5
0.6
0.7
0.8
0.9
1.0
D'
Cambodian (N = 11)
Hezhen (N =9)
Dai (N = 10)
Japanese (N = 29)
Daur (N = 9)
Lahu (N = 10)
Han (N = 44)
Miaozu (N = 10)

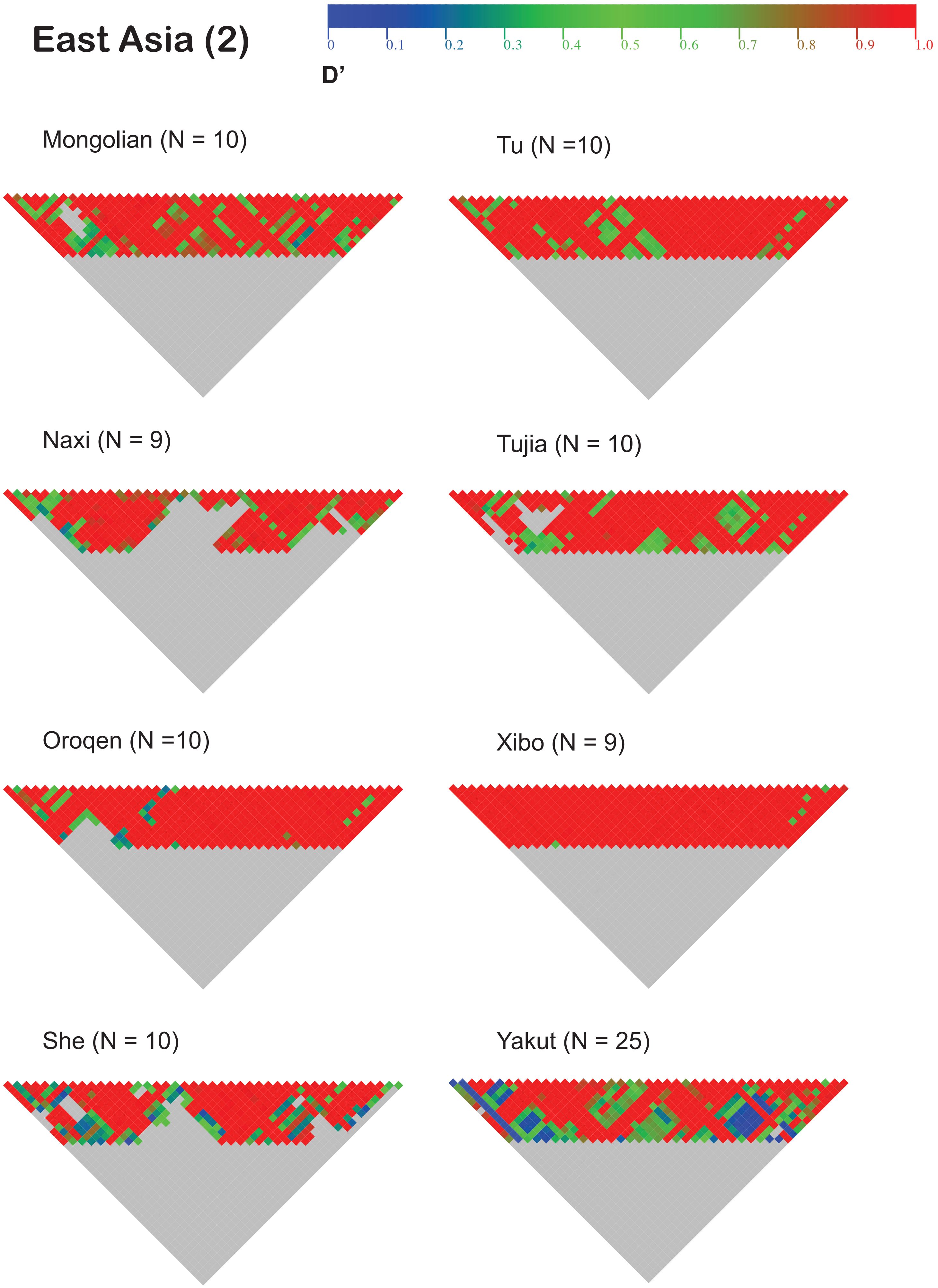
East Asia (2)
0
0.1
0.2
0.3
0.4
0.5
0.6
0.7
0.8
0.9
1.0
D'
Mongolian (N = 10)
Tu (N =10)
Naxi (N = 9)
Tujia (N = 10)
Oroqen (N =10)
Xibo (N = 9)
She (N = 10)
Yakut (N = 25)

# East Asia (3)

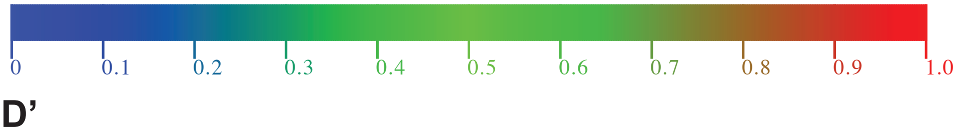


Yizu (N = 10)

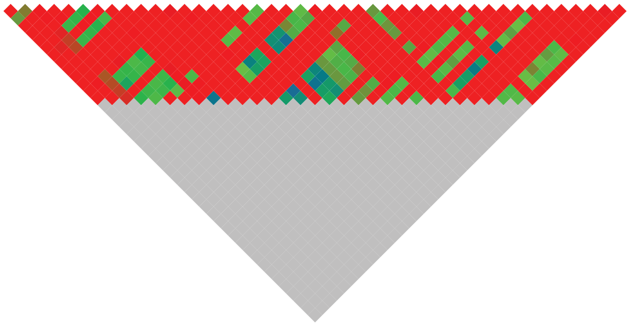

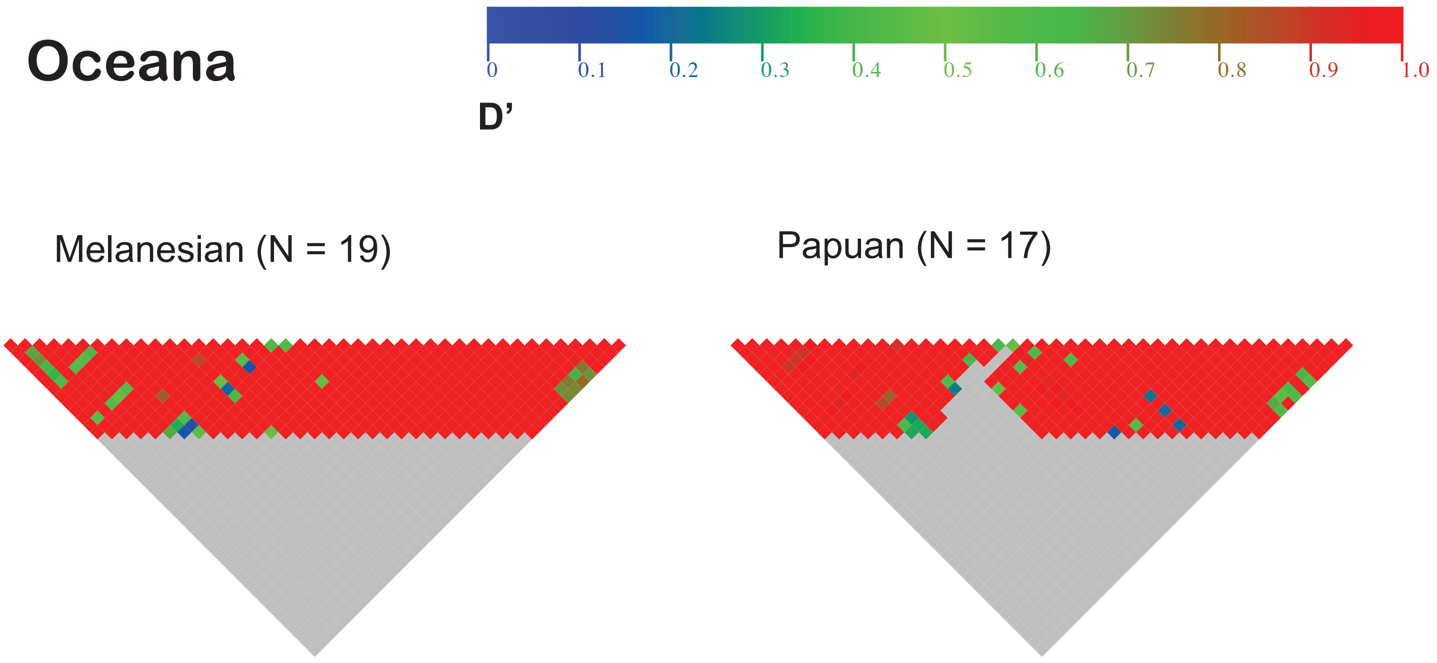
Oceana
0
0.1
0.2
0.3
0.4
0.5
0.6
0.7
0.8
0.9
1.0
D’
Melanesian (N = 19)
Papuan (N = 17)

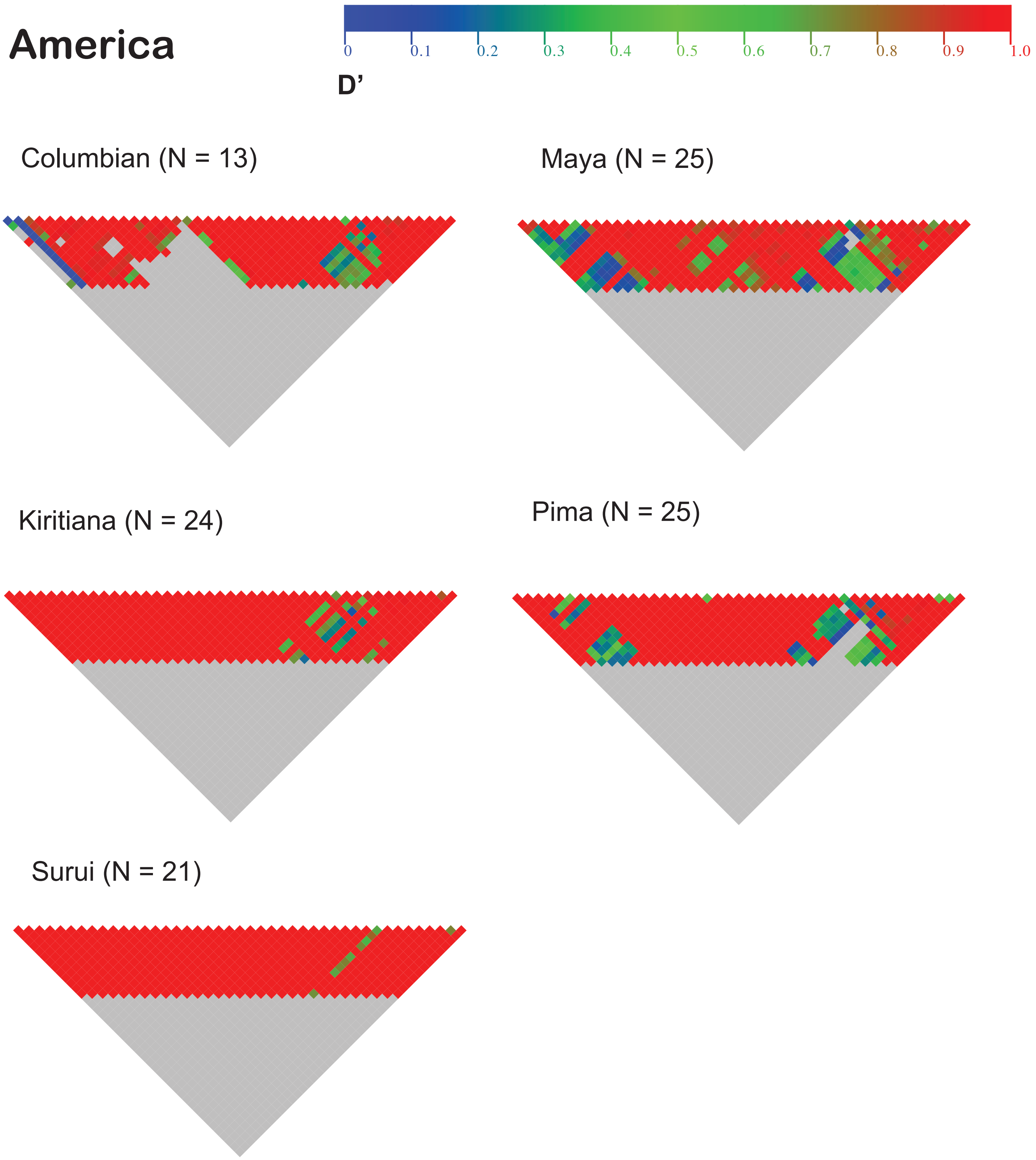
America
D'
0
0.1
0.2
0.3
0.4
0.5
0.6
0.7
0.8
0.9
1.0
Columbian (N = 13)
Maya (N = 25)
Kiritiana (N = 24)
Pima (N = 25)
Surui (N = 21)
